# Oncolytic Bovine Herpesvirus 1 Inhibits Human Lung Adenocarcinoma A549 Cell Proliferation and Tumor Growth by Inducing DNA Damage

**DOI:** 10.3390/ijms22168582

**Published:** 2021-08-10

**Authors:** Wencai Qiu, Xiuyan Ding, Shitao Li, Yongming He, Liqian Zhu

**Affiliations:** 1 Institute of Life Science and Green Development, College of Life Sciences, Hebei University, Baoding 071002, China; wcqiu0909@163.com (W.Q.); yd81843@gmail.com (X.D.); 2 Jiangsu Co-Innovation Center for Prevention and Control of Important Animal Infectious Diseases and Zoonoses, College of Veterinary Medicine, Yangzhou University, Yangzhou 225009, China; 3 College of Life Science and Engineering, Foshan University, Foshan 528231, China; 4 Department of Microbiology and Immunology, Tulane University, New Orleans, LA 70118, USA; sli38@tulane.edu

**Keywords:** BoHV-1, HDACs, TSA, oncolytic virus, virus replication

## Abstract

Bovine herpesvirus 1 (BoHV-1) is a promising oncolytic virus with broad antitumor spectrum; however, its oncolytic effects on human lung adenocarcinoma in vivo have not been reported. In this study, we report that BoHV-1 can be used as an oncolytic virus for human lung adenocarcinoma, and elucidate the underlying mechanism of how BoHV-1 suppresses tumor cell proliferation and growth. First, we examined the oncolytic activities of BoHV-1 in human lung adenocarcinoma A549 cells. BoHV-1 infection reduced the protein levels of histone deacetylases (HDACs), including HDAC1-4 that are promising anti-tumor drug targets. Furthermore, the HDAC inhibitor Trichostatin A (TSA) promoted BoHV-1 infection and exacerbated DNA damage and cytopathology, suggesting a synergy between BoHV-1 and TSA. In the A549 tumor xenograft mouse model, we, for the first time, showed that BoHV-1 can infect tumor and suppressed tumor growth with a similar high efficacy as the treatment of TSA, and HDACs have potential effects on the virus replication. Taken together, our study demonstrates that BoHV-1 has oncolytic effects against human lung adenocarcinoma in vivo.

## 1. Introduction

Bovine herpesvirus type 1 (BoHV-1) is an important viral pathogen of cattle that can induce severe respiratory lesions, conjunctivitis, abortion, vulvovaginitis, balanopostitis and systemic infection in neonate calves [1]. BoHV-1 belongs to the family *Herpesviridae* and subfamily *Alphaherpesvirinae* and shares a number of biological properties with herpes simplex virus types 1 and 2 (HSV-1 and HSV-2) [2]. Diverse HSV-1 strains have been developed as cancer therapeutic reagents by deletion of viral genes essential for replication in normal tissue but not in cancerous cells [3]. Recently, the first oncolytic HSV-1, talimogene laherparepvec (T-VEC, also known as OncoVEXGM-CSF), has been approved for the treatment of melanoma patients with injectable but non-resectable lesions in the skin and lymph nodes in USA, Europe and Australia [4,5,6].

Extensive studies have indicated that BoHV-1 has broad spectrum of anticancer effects, whilst it cannot infect either human body or healthy human cells [7,8,9,10]. Thus, BoHV-1 is safe for cancer therapeutics. Human CD155 that belongs to the nectin-like molecule family has been identified as an entry receptor for BoHV-1 [11,12]. CD155 is overexpressed widely in multiple tumor tissues, including the human lung adenocarcinoma [13]. Moreover, the patients of lung cancer with programmed death-ligand 1(PD-L1) and CD155 expressed at high levels normally have the shortest survival rate [14]. The lung adenocarcinoma cell line A549 with high expression of CD155 is a well-characterized cellular model for studies of lung cancer, and BoHV-1 can productively infect A549 cells and induce cell death partially via induction of DNA damage [7,8,15]. However, whether BoHV-1 inhibits human lung tumor growth and development in vivo is not known.

DNA damage such as DNA double-strand breaks (DSBs) are among the most harmful lesions to cells, as the incorrect repair of just one DSB can consequently lead to chromosome instability and cell death [16]. Cancer chemotherapy and radiotherapy are generally designed to kill cancer cells mostly by induction of DNA damage [17,18]. Actually, it is widely recognized that the most prominent treatment option for cancers is a therapy that can induce DNA damage [18,19].

Chromatin modification plays a pivotal role in DNA damage responses. Increasing evidence has suggested that the histone deacetylases (HDACs) play an essential role in maintaining genomic stability by recruitment of DNA repair associated proteins to the DNA breaks [20]. Therefore, HDAC inhibitors provide a unique avenue for cancer therapy through induction of DNA damage [16,18,21]. Currently, several HDAC inhibitors, such as Trichostatin A (TSA), have been approved by U.S. Food and Drug Administration (FDA) for the treatment of various cancers [22]. TSA could not inhibit BoHV-1 production in Madin-Darby bovine kidney (MDBK) cells [23]. Thus, we hypothesized that combination of TSA with BoHV-1 may lead to enhanced anticancer efficacy.

In this study, we establish BoHV-1 as a novel oncolytic virus with potential for lung cancer therapy. We first found that BoHV-1 infection led to protein depletion of HDACs and induced DNA damage. The HDAC inhibitor TSA facilitated BoHV-1 infection and DNA damage. In the A549 tumor xenograft mouse model, BoHV-1 suppressed tumor growth with a similar high efficacy as the treatment of TSA. However, the enhanced anticancer efficacy was not observed by the combination treatment of BoHV-1 with TSA in A549 cell line derived xenograft mouse model. However, for the first time we showed that the virus could enter and replicate in tumor tissues in vivo detected by immunohistochemistry (IHC).

## 2. Results

### 2.1. BoHV-1 Infection Reduces HDAC Protein Expression

**Figure 1:**
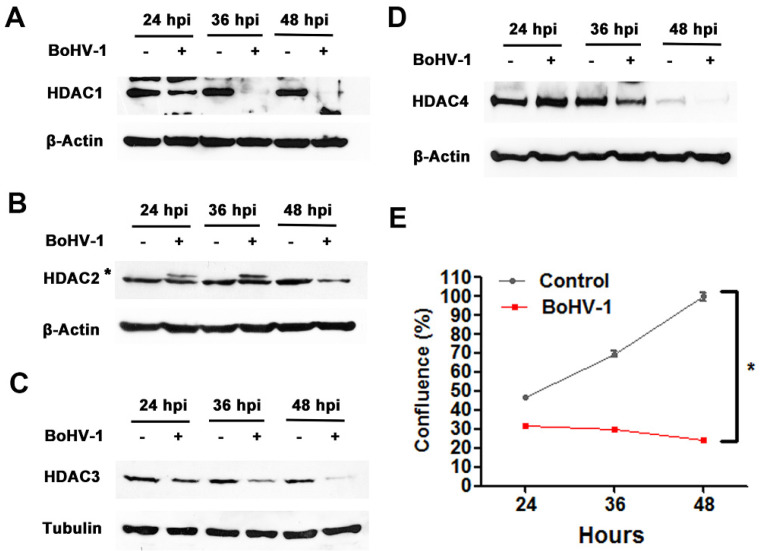
The effects of BoHV-1 infection had on the expression of histone deacetylases (HDACs). (**A**) A549 cells in 60 mm dishes were mock infected or infected with BoHV-1 at an MOI of 0.1 for 24, 36 and 48 h. The cell lysates were then prepared for Western blot to detect the protein expression of HDAC1 (**A**), HDAC2 (**B**), HDAC3 (**C**) and HDAC4 (**D**). Data shown are representative of two independent experiments. (**E**) A549 cells of sub-confluent in 24-well plates were infected with BoHV-1 at an MOI of 0.1. At an indicated time point the cells were collected, stained with trypan blue, counted the numbers of live cells. Cell confluence that indicated growth were calculated as described in materials and methods. Data shown are means of three independent experiments. Statistical difference was determined using a one-way analysis of variance test (ANOVA). * *p* < 0.05.

As BoHV-1 induces DNA damage in cell cultures and HDACs are critical for DNA repair, we suspected that BoHV-1 might affect HDAC protein expression. Thus, we investigated the protein levels of HDAC1, 2, 3 and 4 in A549 cells infected with BoHV-1 over a time course of 24, 36 and 48 h. As shown in Figure 1, the protein levels of all HDACs were gradually decreased with distinct extents in different time points during virus infection. After 48 h, all HDACs were barely detectable (Figure 1A–D), suggesting that BoHV-1 infection gradually reduces HDAC protein expression. In line with above results, the virus infection gradually decreased cell growth relative to the uninfected controls (Figure 1E), suggesting that the cells were infected by virus. Of note, there was an additional upper band (denoted with star) detected by the HDAC2 antibody in cells after infection for 24 and 36 h (Figure 1B). It suggests that viral infection might lead to post-translational modifications of HDAC2, which will be investigated in the future.

**Figure 2:**
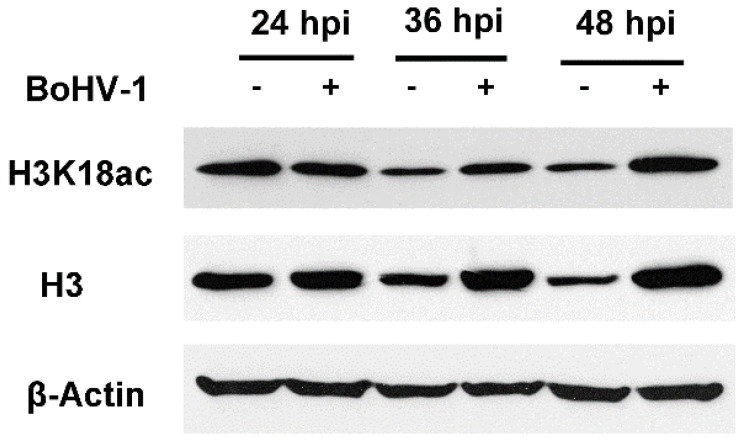
The effects of BoHV-1 infection had on the protein levels of H3K18ac. A549 cells in 60 mm dishes were mock infected or infected with BoHV-1 at a MOI of 1 for 24, 36 and 48 h. The cell lysates were then prepared for Western blot to detect the protein levels of H3K18ac and H3. Data shown are representative of two independent experiments.

HDACs are responsible for histone deacetylation. In line with BoHV-1-induced HDAC protein depletion, the protein levels of acetylated histone 3 (H3) at K18 (H3K18ac) increased at 36 and 48 hpi (Figure 2). In addition, total protein levels of H3 were also increased at 36 and 48 hpi, respectively, supporting the increased accumulation of H3K18ac following virus infection. Taken these data together, BoHV-1 infection in the adenocarcinomic cell line A549 has effects on the protein expression of HDACs, and consequently has influence on the acetylation of H3.

### 2.2. HDACs Inhibitor Trichostatin A (TSA) Promotes BoHV-1 Replication

**Figure 3:**
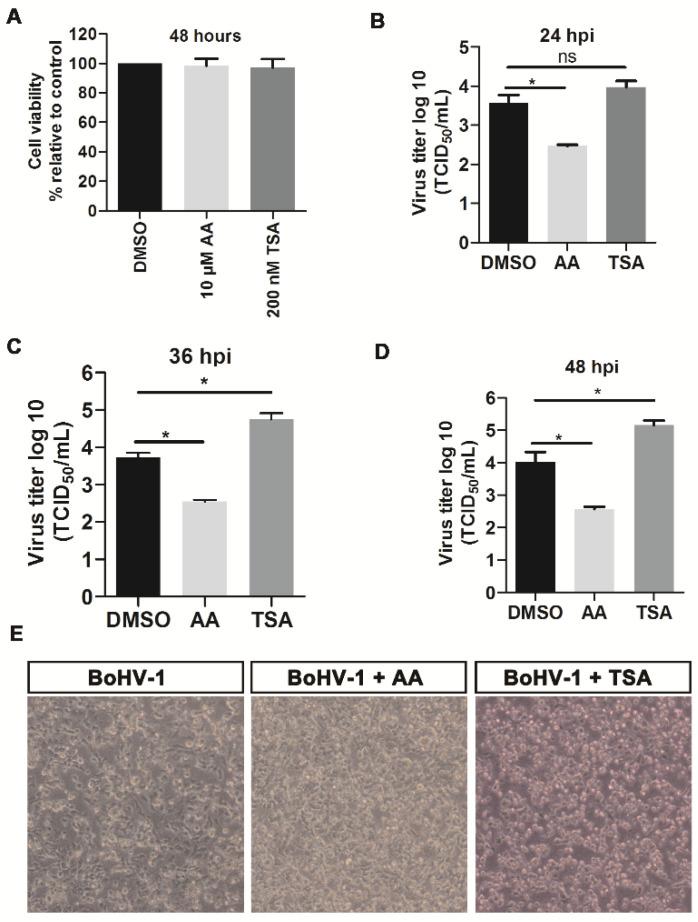
The effects of HDAC inhibitor TSA had on BoHV-1 productive infection in A549 cells. (**A**) A549 cells were treated with DMSO vehicle control, AA (10 μM) and TSA (200 nM) for 48 h, respectively, then the cytotoxicity of individual chemical on the cell culture was determined using Trypan-blue exclusion test. (**B**) A549 cells pretreated with either DMSO vehicle control or HATs inhibitor AA (10 μM), and HDACs inhibitor TSA (200 nM) for 2 h, were infected with BoHV-1 (MOI = 0.1) along with treatment of either AA (10 μM) or TSA (200 nM) for 3 h, then fresh medium containing indicated chemicals were replaced after washing three times. After infection for 24 h (**B**), 36 h (**C**) and 48 h (**D**), the virus productions were determined in MDBK cells, with result expressed as TCID50/mL. Data shown are means ± SD of three independent experiments. Statistical analyses were performed using Student’s *t*-test (* *p* < 0.05). ns, not significant. (**E**) After infection for 48 h, the cell morphology was observed under a light microscope. Data shown are represents of three independent experiments.

Since the virus infection led to protein depletion of HDAC1, 2, 3 and 4, we then examined the roles of histone acetylation in BoHV-1 infection by using two chemical inhibitor TSA and Anacardic acids (AA). TSA inhibits class I and II HDACs [24], while AA inhibits histone acetylation transferases (HAT) [25]. Both AA and TSA at the indicated concentrations did not show obvious cytotoxicity to A549 cells (Figure 3A), suggesting these concentrations are suitable for further experiments. TSA treatment had little effects on virus replication at 24 hpi but increased viral titer by approximately 10-fold compared to the DMSO-treated controls at 36 and 48 hpi (Figure 3C,D). By contrast, AA inhibited BoHV-1 infection and reduced viral titer by more than 10-fold at 24, 36 and 48 hpi (Figure 3B–D), which substantiates that inhibition of histone acetylation enhances viral infection.

Next, the effects of both AA and TSA on the morphology of virus-infected cells were examined at 48 hpi. AA moderately rescued virus infection-induced cytopathology in comparison to the mock-treated virus infected cells. By contrast, virus infection-induced cytopathology was enhanced by TSA (Figure 3E). The enhanced cytopathologic effects (CPE) of virus infection by TSA correlated with the increased virus titers (Figure 3D).

Taken together, these data suggested that HDAC inhibitor, TSA, enhances BoHV-1 productive infection and improves virus infection-induced CPE.

### 2.3. TSA Enhances DNA Damage Induced by BoHV-1 Infection

**Figure 4:**
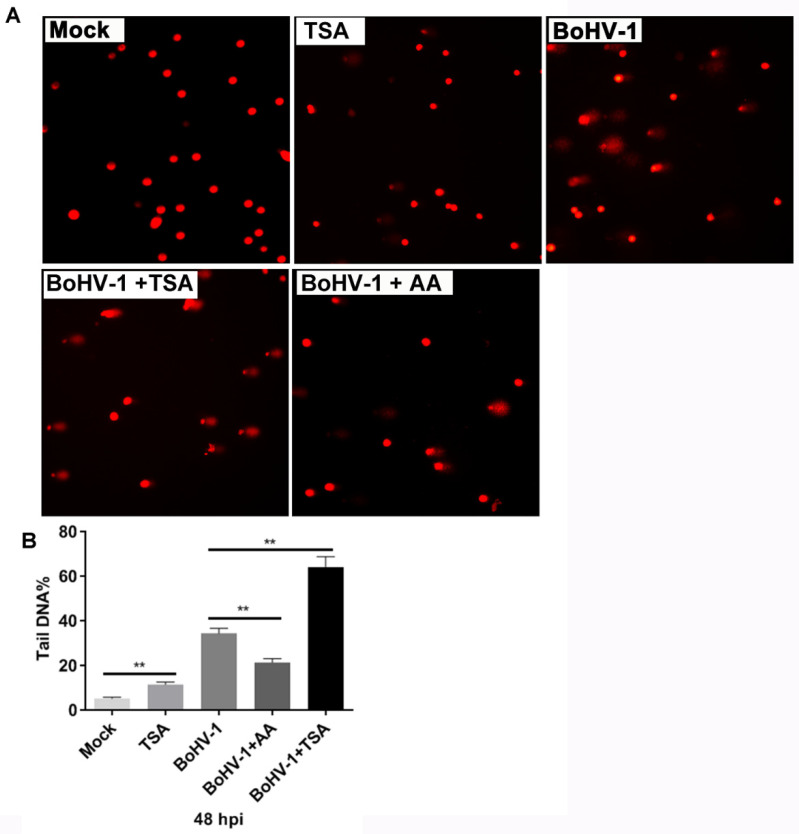
The effects of HDAC inhibitor TSA had on BoHV-1 infection-induced DNA damage in A549 cells. (**A**) A549 cells were infected with BoHV-1 (MOI = 0.1) and treated with either AA or TSA at indicated concentrations, plus a pretreatment for 2 h with indicated inhibitors, respectively. After infection for 48 h, DNA damage in individual cells was determined by using comet assay, the images were acquired under a fluorescence microscope. (**B**) three hundred cells were randomly selected from each sample for the analysis of TailDNA% with software CASP. Data shown are means ± SD of three independent experiments. Statistical analyses were performed using Student’s *t*-test (** *p* < 0.01).

It has been reported that both BoHV-1 and TSA induce oxidative DNA damage in cancer cells [15,26,27], which was confirmed by our data with comet assay, a method to determine the relative intensity of DNA strand breaks in single cells (Figure 4A). We hypothesized that TSA might enhance BoHV-1 infection-induced DNA damage synergistically. As shown in Figure 4A, comet tails were induced at 48 h after infection and additional TSA treatment led to wider and longer comet tails. We further calculated the extent of DNA damage by the ratio of DNA fluorescence in the tail to that in the whole cell (tailDNA%). The tailDNA% was 34.28% in cells infected with BoHV-1 alone. TSA treatment increased the tailDNA% to 64.14%. By contrast, AA treatment reduced the tailDNA% to 21.35% (Figure 4B), partially because of the reduced virus titers by AA. Taken together, these data suggest that TSA enhances BoHV-1-induced DNA damage.

### 2.4. BoHV-1 Suppresses Tumor Growth in A549 Cell Derived Xenograft Mouse Model

**Figure 5:**
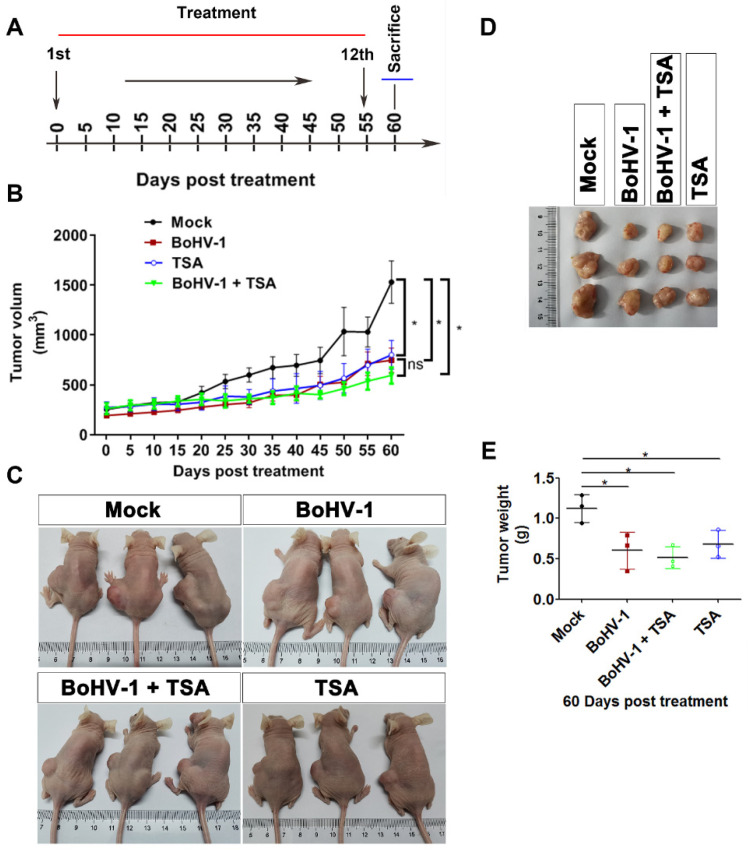
Oncolytic effects of BoHV-1 on human lung adenocarcinomas in A549 xenograft nude mouse model. (**A**) Diagram showing schedule of the treatment. A549 tumor bearing mice were either mock treated with PBS or treated with BoHV-1 (2 × 10^5^ pfu/mouse) TSA (0.5 mg/kg), as well as BoHV-1 and TSA combination, from day 0 to day 55 with intervals of 5 days. At day 60 the mice were sacrificed to collect the tumor tissues. (**B**) Tumor volumes were measured from day 0 to day 60 with intervals of 5 days as indicated the diagram. Data shown in tumor growth curves are mean ± SD (*n* = 3). Statistical difference at day 60 between each group was determined using Student’s *t*-test. * *p* < 0.05; ns, not significant. (**C**) Tumors in the mice were shown at the endpoint of the study. (**D**) Morphology of tumors removed from the mice at the endpoint of the study. (**E**) The volumes of tumors removed from the mice at day 60. Student’s *t*-test was employed to determine the differences between the two groups (* *p* < 0.05).

The findings that TSA enhances BoHV-1 infection and virus infection-induced DNA damage prompted us to investigate whether combination therapy with BoHV-1 and TSA results in a synergistic antitumor efficacy in vivo. The A549 cells derived xenografts in nude mice were adopted. Mice were randomly divided into four groups (*n* = 3), treated with PBS, BoHV-1, TSA or BoHV-1 plus TSA, respectively, at an interval of 5 days as depicted in Figure 5A. At day 60 all the mice were alive without showing ulcerate in the skin. The tumor growth curve indicated that the progression of established tumors was effectively suppressed by monotherapy with either BoHV-1 or TSA with similar efficiency (Figure 5B). Unexpectedly, little synergic inhibitory effects by the combination therapy with BoHV-1 and TSA were found in comparison to individual monotherapy (Figure 5B).

When the tumors were harvested at endpoint (day 60), we found that the tumor sizes were much smaller in groups of either monotherapy or combination therapy when compared to the PBS control group (Figure 5C,D). The average tumor weight of the control group was 1.126 ± 0.172 g, whereas they were 0.602 ± 0.227 g, 0.680 ± 0.171 g and 0.516 ± 0.137 g in the groups with treatment of BoHV-1, TSA and combined BoHV-1-TSA therapy, respectively (Figure 5E). In addition, we noticed that the tumor tissues were much softer in the groups treated by both BoHV-1 and BoHV-1-TSA relative to the PBS control group. These results suggested that monotherapy of BoHV-1 and TSA have similar effects on suppressing tumor growth, but the combinational therapy might not have a synergic effect.

### 2.5. BoHV-1 Infection Induces Tumor Cell Death in Xenografts

**Figure 6:**
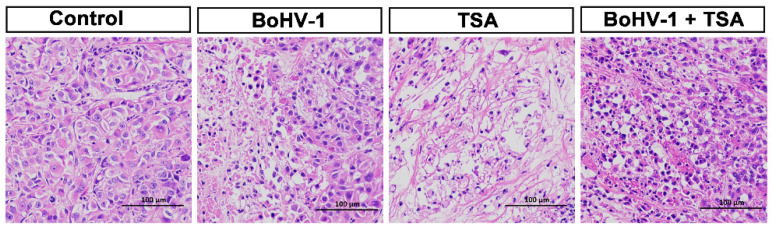
The evaluation of BoHV-1 antitumor effects by using histological analysis. The mice were either mock treated with PBS control, or treated with BoHV-1, TSA, as well as BoHV-1 and TSA combination. Tumor sections from each group were subjected to H&E staining. Images shown were represents of one mouse in each group.

**Figure 7:**
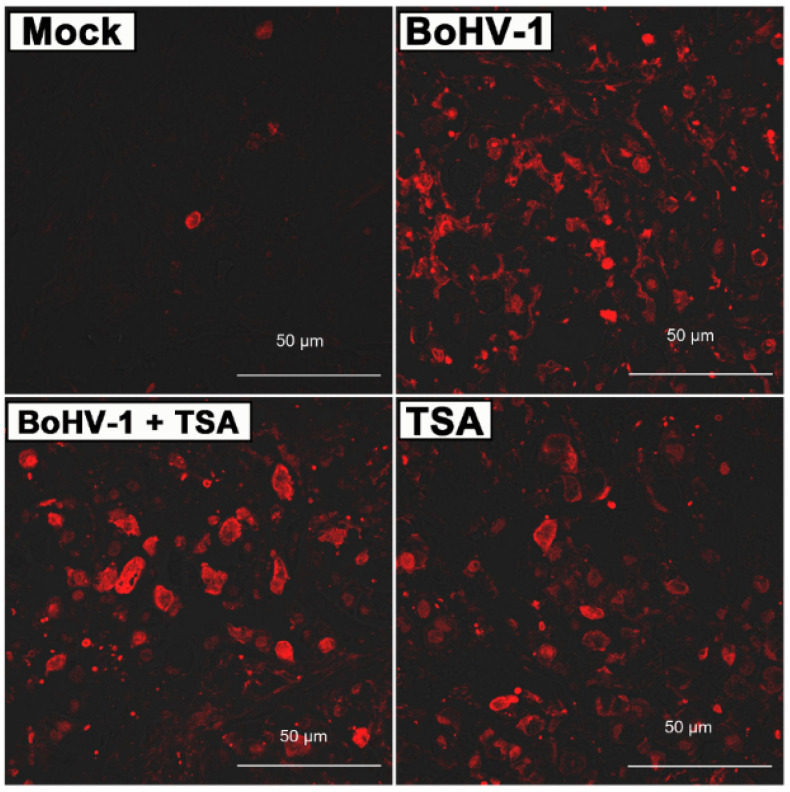
Of died cells in tumor sections. Tumor sections from PBS mock treated, BoHV-1, TSA, as well as BoHV-1 and TSA treated mice were processed for TUNEL assay. Images shown were represents of one mouse in each group.

To further assess the antitumor activities, we performed histological analyses of the harvested tumors. H&E staining found apoptotic cells, indicated by darkly stained and condensed chromatin, in the tumors treated with BoHV-1, TSA or the combination treatment (Figure 6). Conversely, only few apoptotic cells were found in the tumors treated with PBS. Furthermore, terminal deoxynucleotidyl transferase mediated dUTP nick end labeling (TUNEL) assay was performed to determine apoptotic and necrotic cells by detection of DNA breaks. Similar to the H&E staining, the TUNEL+ cells were barely detected in tumors treated with PBS but readily detected in the tumors of other groups (Figure 7). However, the BoHV-1-TSA combination therapy induced a similar number of TUNEL+ cells as the monotherapy using BoHV-1, and TSA alone (Figure 7). These data suggest that BoHV-1 has oncolytic activity towards human lung adenocarcinoma tumor in xenografts model.

**Figure 8:**
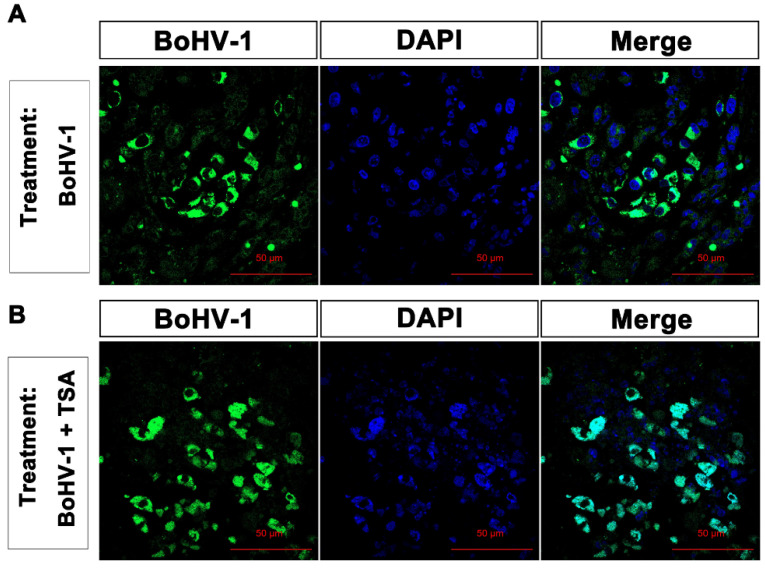
The detection of virion-associated proteins in the tumor sections with IFA. Tumor sections treated by BoHV-1 (**A**) and BoHV-1-TSA (**B**) were processed for the detection of virion-associated proteins with immunohistochemistry by using the anti-BoHV-1 serum. Images shown were represents of each group.

Though it has been reported that BoHV-1 is a promising oncolytic virus, whether it is able to infect tumors in vivo has not been reported. In this study, the virus infection was measured by detection of the virion-associated proteins through immunohistochemistry (IHC) assay using the anti-BoHV-1 antiserum. Representative IHC data from studies of tumors treated with either BoHV-1 or combined BoHV-1-TSA is shown in Figure 8A,B, respectively. Staining of virion-associated proteins was detected in the tumors treated by either BoHV-1 (Figure 8A), or combined BoHV-1-TSA (Figure 8B). Apoptotic bodies were concomitantly detected in the cells with positive staining of virion-associated proteins in tumors (Figure 8). Interestingly, most of the virion-associated proteins located at the cytoplasm but not nucleus in the tumor cells receiving BoHV-1 monotherapy (Figure 8A). In contrast, they were mainly located at the nucleus but not cytoplasm in the tumor cells following combined treatment of BoHV-1-TSA (Figure 8B), suggesting that HDACs are potentially associated with the localization of virion-associated proteins. Either condensed or segregated chromatin, and apoptotic bodies were observed in the tumor cells harboring virion-associated proteins (Figure 8), suggesting that either virus infection or replication in the tumor could induce apoptosis.

## 3. Discussion

Generally, post-translational modifications of histones and DNA would lead to alteration of chromatin structure. Histone acetylation is one of the best-characterized histone modifications [28]. Accumulating studies have indicated that acetylation of histones is implicated in the infection of herpesviruses by affecting either viral gene transcription or viral DNA replication [29]. For example, partial HSV-1 genome is dynamically associated with modified histones with distinct patterns during latent and lytic infection either in vitro or in vivo [28,30,31,32]. Interestingly, the protein levels of acetylated H3, such as H3K18ac were increased by BoHV-1 infection in A549 cells (Figure 2), but decreased in MDBK cells [23], suggesting that acetylation of histones, such as H3, may be affected by virus infection with cell-type dependent patterns.

It is well established that both HATs and HDACs are involved in the acetylation of histones with contradictory effects. Together with our previous report, we found that HATs and HDACs play different roles in virus productive infection because HATs inhibitor AA is able to inhibit virus productive infection in both MDBK cells [23] and A549 cells (Figure 4), while HDACs inhibitor TSA increases virus production in A549 cells (Figure 4) but not in MDBK cells [23], suggesting that HDACs are potential host factors limiting the virus productive infection in A549 cells. To our knowledge this is the first identified compound that can increase BoHV-1 production in vitro. These data suggested that HDACs are potential cellular factors limiting the virus replication in A549 cells. Moreover, a variety of HDAC inhibitors, such as TSA, are implicated in cancer therapeutic, partially through preventing DNA damage repair [33,34]. Considering this evidence, we expected that combination therapy with BoHV-1 and TSA may get synergism of antitumor efficacy. Unexpectedly, synergic effects of combined therapy were not obtained in the treatment of A549 tumors of xenografts. Mono treatment by using BoHV-1 showed almost the same effects as BoHV-1-TSA combination as evaluated by the tumor size, tumor weight, histologic examination and TUNEL assay (Figure 5, Figure 6 and Figure 7). Obviously, the antitumor effects of combination therapy with BoHV-1-TSA in vitro are largely different from that in vivo. However, it has been reported that BoHV-1 oncolytic activity is enhanced when applied in combination with azacytidine, an FDA approved drug for cancer therapy, as assessed in tolerized cotton rat model harboring breast adenocarcinoma [35], raising a succeed example that the virus oncolytic efficacy could be enhanced by a given compound. Maybe it is also plausible to find a compound with capacity to enhance virus oncolytic efficacy for the treatment of human lung adenocarcinoma in vivo, which need further studies in the future.

The oncolytic efficacy of BoHV-1 has been extensively characterized in cotton rat model of breast adenocarcinoma [35,36]. Here, our data indicated that the virus also has capacity to induce cell death and suppressing growth of lung adenocarcinoma as assessed in the A549 cell derived xenografts (Figure 5, Figure 6 and Figure 7). However, whether the virus can enter tumor cells and accomplish replication cycles in vivo has not been reported. In this study, for the first time we showed that virion-associated proteins can be readily detected in the tumors with IHC by using a polyclonal antibody against virion-associated proteins (Figure 8). Of note, this polyclonal antibody was generated by immunization of purified viral particles. So, the part of the virion-associated proteins detected by IHC may represents of viral particles. It is known that BoHV-1 is a nuclear virus. Once entering the cytoplasm of infected cells, the viral capsids move toward the nuclei, where de novo viral particles are subsequently released into extracellular environment by passing through cytoplasm. Here, we found that virion-associated proteins were mainly detected in the cytoplasm in the tumor tissues receiving BoHV-1 monotherapy (Figure 8A). In contrast, they were mainly detected in the nucleus received treatment by BoHV-1-TSA (Figure 8B). Obviously, they did not match the canonical theory. They may represent unreported mechanisms of virus replication exclusively existed in the cancer tissues, which warrants extensive studies in the future. In addition, we could not exclude the possibility that the IHC positive staining may represent replication incompetent viruses. If so, our results will validate a previous report that BoHV-1 could also elicit tumor cell death in the absence of a productive infection [7], because virion-associated proteins were detected in populations of apoptotic bodies (Figure 8). Taken together, for the first time we showed that BoHV-1 could enter tumor cells in A549 cell derived xenograft model.

As a potential oncolytic vector BoHV-1 has the following advantages over HSV-1: (i) the virus infection in human tumor cells fails to elicit interferon (IFN) production [8]. (ii) it has been reported that most tested human antibodies or serum samples having HSV-1 neutralizing capacity fails to neutralize BoHV-1 infection [8]. (iii) unlike HSV-1 that can infect human and establish latency which may lead to concurrent diseases, BoHV-1 could not infect human as well as human healthy cells, but only the tumor cells [8]. So BoHV-1 is safe for our human, and it is possible that the virus could escape the immune responses induced by HSV-1 infection potentially existed in the patient. While they may restrict HSV-1 infection. These advantages may allow BoHV-1 to be a safe and efficient oncolytic vector for systemic treatment of diverse tumors. In this study, we noticed that virion-associated proteins could only be detected in limited areas but not in the whole tumor sections with IHC. We speculated that the virus is transmitted with low efficiency in the tumor tissue, which may limit its antitumor efficacy. While BoHV-1 still has oncolytic effects against human lung adenocarcinoma as evaluated by using nude mouse model, which supports the previous report that regardless of productive infection or not, BoHV-1 exposure is able to elicit tumor cell death in long-term culture even at low infection dose [7,36]. Thus, in addition to productive infection, immunomodulation within the tumor microenvironment also plays important role in mediating the antitumor effects, which need further studies to elucidate the mechanism in the future. However, we suggested that generating a virus with increased transmission capacity and productive infection is an effective approach to enhance the antitumor effects.

## 4. Materials and Methods

### 4.1. Cells and Virus

A549 cells (Chinese Model Culture Preservation Center, Shanghai, China) were maintained in DMEM supplemented with 10% fetal bovine serum (cat#10270-106) (Thermo Fisher Scientific, Waltham, MA, USA). MDBK cells (Chinese model culture preservation center, Shanghai, China) were maintained in DMEM supplemented with 10% horse serum (cat# S9050) (Solarbio, Beijing, China). BoHV-1 isolate NJ-16-1, isolated from bovine semen samples in China [37], was used in this study. The virus was propagated in MDBK cells. Aliquots of virus stocks were stored at −70 °C until use.

### 4.2. Antibodies and Reagents

The following primary antibodies were used in this study: HDAC1 (histone deacetylas 1) mouse monoclonal antibody (mAb) (cat#5356, 1:1000), HDAC2 mouse mAb (cat#5113, 1:1000), HDAC3 mouse mAb (cat #3949, 1:1000), HDAC4 rabbit mAb (cat #7628, 1:1000), Histone H3 rabbit mAb (cat#4499, 1:1000), Acetyl-Histone H3 (Lys18) (H3K18ac) rabbit mAb (cat#13998, 1:1000), β-Actin rabbit mAb (cat#4970, 1:1000), HRP (horseradish peroxidase), labeled anti-mouse IgG (cat#7076, 1:3000) and HRP labeled anti-rabbit IgG (cat#7074, 1:3000), were all purchased from Cell Signaling Technology (Beverly, MA, USA). β-Tubulin rabbit polyclonal antibody (cat# AC015) was purchased from Abclonal Science Inc (Woburn, MA, USA). The HAT inhibitor inhibitors, including Anacardic acid (AA) [41A7236], as well as HDAC inhibitor trichostatin A (TSA) (cat#8552), were ordered from Sigma-Aldrich (St. Louis, MO, USA). Goat anti-BoHV-1 serum (cat# PAB-IBR) was purchased from VMRD Inc (Pallman, WA, USA). Alexa Fluor 488^®^-conjugated donkey anti-goat IgG H&L (ca# ab150129) was purchased from Abcam (Cambridge, England).

### 4.3. Cell Viability Assay

Cell viability was assessed according to the method described elsewhere with modification [38,39]. In brief, A549 cells of monolayer in 96-well plates (Corning Costar, Corning, NY, USA) were exposed to the assigned inhibitors of either AA or TSA at indicated concentrations for 48 h. After treatment with trypsin and trypan blue staining, the cells were collected and counted under microscope. The percentage of cell viability in the chemical treatment groups was calculated by normalization of the number of live cells to that in the control samples. The cell viability of mock treated control was arbitrarily assigned as 100%.

### 4.4. Virus Replication Inhibition Assay

A549 cells of monolayer in 24-well plates were pretreated with either DMSO control or indicated inhibitors of either AA or TSA indicated concentrations for 2 h. After pretreatment the cells were infected with BoHV-1 (MOI = 0.1) in the presence of DMSO control or indicated inhibitors for 3 h. The cells were then extensively washed three times using PBS (PH, 7.4), and fresh DMEM medium with or without chemicals were replaced. After infection for 24, 36 and 48 h, the cell cultures were collected and subjected to frozen-thawing twice, then virus titers were determined in MDBK cells. The results were expressed as TCID50/mL calculated using the Reed–Muench method.

### 4.5. Western Blotting Analysis

A549 cells in 60 mm dishes were mock infected or infected with BoHV-1 at an MOI of 0.1 for 24, 36 and 48 h. Cells lysates were prepared with RIPA buffer (1 × PBS, 1% NP-40, 0.5% sodium deoxycholate and 0.1% SDS) supplemented with protease inhibitor cocktail, and cleared by centrifugation at 13,000 rpm for 10 min at 4 °C. The supernatant was subjected to Western blotting analysis using the designated antibodies. In parallel, either β-Actin or β-Tubulin was probed as a protein loading control.

### 4.6. Comet Assay

DNA damage was evaluated through alkaline comet assay (single cell gel electrophoresis) according to the method described elsewhere with modification [40]. In brief, A549 cells of monolayer in 24-well plates were pretreated with either DMSO control or indicated inhibitors of either AA or TSA at indicated concentrations for 2 h. The cells were infected with BoHV-1 (MOI = 0.1) in the presence of either DMSO control or indicated inhibitors for 3 h. The cells were then washed three times using PBS (pH 7.4), and fresh DMEM medium with or without chemicals were replaced. After infection for 48 h, the cells were collected and suspended in low melting agarose placed on slides coated with 1% normal melting agarose, and low melting agarose was then added as the top layer. Cells were lysed in cold (4 °C) lysis buffer (2.5 M NaCl, 100 mM Na2EDTA, 10 mM Tris, 1% Triton X and 10% DMSO, pH 10.0) for 1 h. The slides were subjected to horizontal gel electrophoresis in cold (4 °C) alkaline electrophoresis buffer (300 mM NaOH and 1 mM Na2EDTA, pH 12.5) at 25 V and 300 mA for a 40 min. The slides were then soaked twice with neutralization buffer (0.4 M Trizma base, pH 7.5, 4 °C) for 10 min and air-dried. DNA was stained with PI (20 μg/mL) and images were subsequently captured by using a fluorescence microscope. Approximately three hundred cells from each sample were analyzed using CASP software (University of Wroclaw, Worsaw, Poland). Percentage of the DNA tail (tailDNA%) was used as the metric for DNA damage [40,41].

### 4.7. Assessment of BoHV-1 Oncolytic Efficacy by Using A549 Cell Derived Xenograft Mouse Model

Female BALB/c nude mice of 4-week old were purchased from animal facility of Hebei University (Baoding, Hebei Province, China). All procedures complied with the Chinese National Institutes of Health Guidelines for the Care and Use of Laboratory Animals. Ethical approval for the study was granted by the Ethics Committee of College of Life Science in Hebei University. Each mouse was subcutaneously injected approximately 1 × 10^7^ A549 cells to induce tumor development. When the tumor reached an average volume of approximately 5–10 mm in diameter, the mice were randomly divided into different four groups, and receive the following treatments: 2 × 10^5^ PFU of BoHV-1(BoHV-1 group, *n* = 3), 0.5 mg/kg of TSA (TSA group, *n* = 3) and BoHV-1 together with TSA (BoHV-1 + TSA group, *n* = 3), as well as PBS as a control (control group, *n* = 3), respectively, via intratumoral inoculation. For an interval of five days, the mouse received one treatment. Tumor growth was monitored every 5 days. Tumor sizes were measured in two dimensions with a caliper and calculated as described elsewhere [36]. After treatment for 60 days, the mice were sacrificed and tumor tissues were harvested, weighed, fixed with 10% formaldehyde and embedded in paraffin. The tumor sections (5 μm) were subjected to hematoxylin-eosin (H&E) staining, TUNEL assay and immunohistochemistry.

### 4.8. Detection of Cell Death with TUNEL Assay

Terminal deoxynucleotidyl transferase mediated dUTP nick end labeling (TUNEL) assay can identify DNA fragmentation, a characteristic of both apoptotic as well as necrotic cells. The cell death in the tumor tissues induced by different treatment was determined by performing TUNEL assay. The study was performed by using the TUNEL assay kit (Beyotime Biotechnology, Shanghai, China, cat# C1090), following the manufacturer’s instructions.

### 4.9. Immunohistochemistry to Detect Virion-Associated Proteins

Tissue sections were deparaffinized with xylene and rehydrated through a series of graded ethanol. For antigen unmasking, sections were immersed in 10 mM sodium citrate buffer (pH 6.0) and heated for 10 min in a microwave. Endogenous peroxide was quenched in 1% hydrogen peroxide for 10 min. The sections were blocked by incubation with 5% normal goat serum for 1 h at room temperature. The antibody directed against BoHV-1 virion-associated proteins was diluted 1:500, added to the slide and incubated at 4 °C overnight. Alexa Fluor 488^®^-conjugated donkey anti-goat IgG H&L was used for secondary antibody binding. The nuclei were stained with DAPI contained in the mounting medium. Immunostaining was observed under the confocal fluorescent microscope.

## 5. Conclusions

In this study, we found that HDACs was potentially involved in BoHV-1 infection, and HDACs inhibitor could enhance the virus production, virus infection-induced DNA damage and CPE, in the lung adenocarcinoma A549 cell culture. BoHV-1 application alone could significantly suppress tumor growth with efficacy identical to the chemical TSA as assessed in A549 xenograft nude mouse model. However, the antitumor efficacy of BoHV-1 was not enhanced by TSA. Interestingly, subcellular localizations of the virion-associated proteins were largely different between monotherapy with BoHV-1 and combined therapy with BoHV-1-TSA, suggesting that HDACs is potentially involved in the virus infection in vivo. Moreover, these findings provide an evidence that the virus could infect the tumor tissues with distinct mechanisms in a context with or without HDAC inhibitor, TSA, providing a novel aspect to reveal the virus replication mechanisms in tumors in vivo, which warrants further studies in the future. In summary, BoHV-1 is a promising candidate for the treatment of lung adenocarcinoma.

## Data Availability

The authors declare that all the data are available upon request.
